# PROMISE international; a clinical post marketing trial investigating the percutaneous deep vein arterialization (LimFlow) in the treatment of no-option chronic limb ischemia patient

**DOI:** 10.1186/s42155-019-0067-z

**Published:** 2019-07-31

**Authors:** Michiel A. Schreve, Michael Lichtenberg, Çagdas Ünlü, Daniela Branzan, Andrej Schmidt, Daniel A. F. van den Heuvel, Erwin Blessing, Marianne Brodmann, Vincent Cabane, William Tan Qing Lin, Steven Kum

**Affiliations:** 1 Department of Vascular Surgery, Northwest Clinics, Alkmaar, the Netherlands, Wilhelminalaan 12, 1815 JD Alkmaar, the Netherlands; 2 Vascular Centre Arnsberg, Arnsberg Clinic, Arnsberg, Germany; 3 Standort Karolinen-Hospital, Stolte Ley 5, 59759 Arnsberg, Germany; 4 Department of Vascular Surgery, University Hospital Leipzig, Leipzig, Germany; 5 Universitätsklinikum Leipzig AöR, Liebigstraße 20, 04103 Leipzig, Germany; 6 Department of Angiology, University Hospital Leipzig, Leipzig, Germany; 7 Department of Radiology, St. Antonius Hospital, Nieuwegein, the Netherlands; 8 Ziekenhuis Nieuwegein, Koekoekslaan 1, 3435 CM Nieuwegein, the Netherlands; 9 Department of Interventional Angiology, SRH Klinikum Karlsbad-Langensteinbach, Karlsbad, Germany; 10 SRH Klinikum Karlsbad-Langensteinbach GmbH, Guttmannstraße 1, 76307 Karlsbad, Germany; 11 Angiologie, Medizinische Universtität Graz, Graz, Austria; 12 Medizinische Universtität Graz, Auenbruggerplatz 27, 8036 Graz, Austria; 13 LimFlow SA,Paris, France, 95 Boulevard Pereire, 75017 Paris, France; 14 Department of Surgery, Vascular Service, Changi General Hospital, Changi, Singapore; 15 Changi General Hospital Pte Ltd, 2 Simei Street 3, Singapore, 529889 Singapore

## Abstract

**Background:**

Critical limb ischemia (CLI) is the clinical end stage of peripheral artery disease and is associated with high amputation, mortality rates and poor quality of life. For CLI patients with no revascularization options, venous arterialization could be an alternative technique for limb salvage. A systematic review and meta-analysis published in 2017 concluded that venous arterialization may be considered a viable alternative. A recent development, is the Percutaneous Deep Vein Arterialization (pDVA), that is CE-marked and currently under investigation of the FDA. This procedure, called LimFlow, is a novel, minimally invasive, endovascular approach to perform a venous arterialization procedure. The limited evidence for its use necessitates a scientific judgement of the pDVA. Therefore, we initiated a prospective clinical post market trial to investigate the outcome of the pDVA in no-option critical limb ischemia.

**Methods/design:**

The objective of this prospective study is to collect *“real-life”* clinical data among a population of patients treated with the pDVA in order to evaluate the clinical effectiveness and safety of the LimFlow System in patients with no-option critical limb ischemia. This study is a single-arm, open-label, prospective, post-market follow-up study to be conducted on up to fifty (50) eligible patients with a twelve-month follow-up period. The Primary endpoint is measured by amputation free survival. Secondary endpoints are complete wound healing, primary and secondary patency, limb salvage, renal function and technical and procedural success. Patients will be assessed at regular intervals during one year after the initial percutaneous deep vein arterialization procedure through clinical evaluation and self-completed questionnaires.

**Discussion:**

The last decade several studies have been published with promising results and the number of treated patients has considerably grown. Venous arterialization could be a valuable treatment option in patients with often no other options than amputation of the affected limb.

The first results in men are promising although more research and long term follow up is needed to establish the efficacy of this new treatment modality.

With this prospective study, we evaluate the clinical effectiveness and safety in patients with no-option CLI treated with the pDVA (LimFlow System).

**Trial registration:**

NCT03321552.

## Background

Critical limb ischemia (CLI) is the clinical end stage of peripheral artery disease (PAD) and is associated with high amputation, mortality rates and poor quality of life. (Sprengers et al., 2010) It is estimated that 5 to 10% of patients with peripheral artery disease older than 50 years will develop severe or critical limb ischemia (CLI) within 5 years and mortality rates are 20% at 6 months after onset. (Norgren et al., 2007) Bypass surgery and more recently endovascular interventions with angioplasty and stenting have become the treatment of choice in order to prevent amputation and resolve rest pain. Endovascular interventions carry lower risk of morbidity and mortality and are often used as the first-line approach in preference to open surgery. (Adam et al., 2005; Katib et al., 2015)

Advancements in endovascular technology and technique have led to high technical success rates; however, failure may occur due to the absence of distal target vessels, severe calcification, and heavy plaque burden that results in elastic recoil and early restenosis after angioplasty. Advanced disease with occlusion of the pedal arteries or only small artery’s (the “desert foot”) represents an end-stage pathology that commonly leads to failure of all revascularization attempts and culminates in major amputation. This is termed no-option CLI.

In the last decade, new treatment options have been explored as for patients with no-option CLI. These include stem cell therapy, spinal cord stimulation and prostanoids therapy. A meta-analysis of placebo controlled trials showed no advantage of stem cell therapy on the primary outcome measures of amputation, survival, and amputation free survival in patients with CLI. (Peeters Weem et al., 2015) Another meta-analysis showed no benefit for prostanoids treatment or other medical treatments. (Abu Dabrh et al., 2015) A Cochrane review concluded that there may be some benefit with spinal cord stimulation for prevention of amputation, however evidence is considered as very low grade, mainly due to imprecision and increased risk of bias. (Ubbink & Vermeulen, 2005)

For CLI patients with no revascularization options, venous arterialization could be an alternative technique for limb salvage. The concept of using the disease-free venous bed as an alternative conduit for perfusion of the peripheral tissues with arterial blood was first published by Halstead and Vaughan in 1912. (Halstead & Vaughan, 1912) Flow in existing collateral vessels will increase, reversal of flow all the way through the capillaries improves tissue nutrition (Ozek et al., 1997) and possibly stimulates angiogenesis. (Baffour et al., 1988)

A systematic review and meta-analysis published in 2017 concluded that venous arterialization may be considered a viable alternative before major amputation. (Schreve et al., 2017) Nevertheless, this technique is not widely being used. This could be due to the low quality of studies together with the fact you have to comprehend with wounds on the foot when performing a classic venous arterialization.

A recent development is the Percutaneous Deep Vein Arterialization (pDVA) that is CE-marked and currently under investigation of the FDA. This procedure, called LimFlow, is a novel, minimally invasive, endovascular approach to perform a venous arterialization procedure. A major advantage of this approach, is the minimal invasiveness with lower periprocedural risks and no creations of wounds in an already critically ischemic leg. The first in men results have recently been published, demonstrating safety and feasibility of the pDVA, with promising early clinical outcome in this small cohort. Long term follow up is needed to establish the efficacy of this new treatment modality. (Kum et al., 2017)

The limited evidence for its use necessitates a scientific judgement of the pDVA. Therefore, we initiated a prospective clinical post market trial to investigate the outcome of pDVA in no-option critical limb ischemia.

## Methods/design

### Objective

The objective of this prospective study is to collect *“real-life”* clinical data among a population of patients treated with the pDVA (LimFlow System) in order to evaluate the clinical effectiveness and safety of the LimFlow System in creating a below-the-knee arterio-venous fistula for venous arterialization in subjects with critical limb ischemia.

Our hypothesis is that in patients with no-option critical limb ischemia, a treatment with pDVA is a feasible, safe, and clinically effective approach. This study is a single-arm, open-label, prospective, post-market follow-up study to be conducted on up to fifty (50) eligible patients with a twelve-month follow-up period. This study was designed and is to be conducted in compliance with the ISO 14155:2011 standard. Outcome is primarily measured as amputation free survival. Results of the LimFlow System risk assessment indicated that device-related risks had been reduced to levels as low as reasonably practicable and comparable with the state of the art. However, although the risks associated with the use of the LimFlow System were comparable with the state-of-the-art, the clinical experience was insufficient to completely characterize the nature and incidence of device-related procedural complications or late clinical complications. The safety and clinical effectiveness has not been evaluated in large cohort. Therefore, post-market clinical follow-up was indicated to more precisely assess the clinical effect and incidence of potential risks or to confirm that their prevalence lied below the threshold of concern and that the safety and clinical outcome is good.

### Study population

Inclusion criteria:

1. Subject must be > 21 and < 95 years of age
Clinical diagnosis of symptomatic critical limb ischemia, defined as Rutherford category 5 or 6
Assessment that no conventional surgical or endovascular treatment is possible
Proximally, the target in-flow artery at the cross-over point must be treatable with a 3.5–4.0 mm stent after pre-treatment (by visual estimate), and be < 50% stenosis
Subject is willing and has adequate support to comply with protocol requirements, including medication regimen and follow-up visits

Exclusion criteria:

1. Concomitant hepatic insufficiency, deep venous thrombus in target limb, uncorrected coagulation disorders, or current immunodeficiency disorder
2. Life expectancy less than 12 months
3. Patient currently taking coumarin/warfarin which, in the opinion of the attending physician, interferes with the patient’s treatment
4. Any significant medical condition which, in the attending physician’s opinion, may interfere with the patient’s optimal treatment
5. Patient currently participating in another investigational drug or device study that has not completed the primary endpoint or that clinically interferes with the endpoints of this treatment
6. Patient unable to give consent
7. Pregnant or breastfeeding women
8. Documented myocardial infarction or stroke within previous 90 days
9. Patients suffering from renal insufficiency (GFR value less than 30 ml/min/1.73 m^2^) who are not on hemodialysis
10. Patients with vasculitis and/or untreated popliteal aneurysms
11. Patients with acute limb ischemia
12. Prior peripheral arterial bypass procedure above or below the knee which could inhibit proximal inflow to the stent graft
13. Lower extremity venous disease with significant oedema in the target limb that may inhibit the procedure and/or jeopardize wound healing, in the investigator’s opinion
14. Known or suspected systemic or severe infection (e.g., WIfI foot Infection grade of 3)
15. Known or suspected allergies or contraindications to stainless steel, nickel, or contrast agent that cannot be adequately pre-treated, or patients who cannot receive anticoagulation or antiplatelet aggregation therapy
16. Severe heart failure, which in the opinion of the investigator may compromise subject’s ability to safely undergo a percutaneous procedure (e.g., known ejection fraction of < 40%, NYHA Classification III-IV)

## Procedure

### Device description

The LimFlow System comprises the following five (5) components:

#### Ultrasound system

The LimFlow ultrasound system consists of a power supply, a laptop computer, and a transceiver box. The system produces a short electrical pulse which is applied to the transmit catheter. The signal received by the receive catheter is amplified, filtered, and digitized. The received signal is then displayed on the laptop as a waveform, giving a visual display of the strength of the received pulse and hence permitting orientation of the two catheters. Software running on the laptop permits the gain of the receiver and other parameters to be adjusted.

#### Extension cable set

The LimFlow extension cables carry power between the LimFlow ultrasound system and the LimFlow arterial and venous catheters.

#### Arterial and venous catheter set

The arterial ultrasound catheter is a 6.5-Fr catheter with a usable length about 100 cm. It is placed over a standard 0.014″ guide wire through a sheath in the femoral artery and advanced to the tibial artery up to the point of total arterial occlusion. The arterial ultrasound catheter has two functions:

1. Locating neighbouring veins: a small single directed ultrasonic transmitter as its tip allows to detect the venous catheter in a neighbouring vein. The handle design and the catheter body allow for an easy torque and push-pull to find the correct location.
2. Connecting to a neighbouring vein by advancing a crossing-needle: the catheter has a handle with a pusher ring, which advances the crossing needle from artery to vein. The catheter is placed into the artery with the needle retracted inside the catheter shaft. A standard 0.014″ guide wire can be placed through the advanced needle from the proximal hub (this wire is referred to as the *“crossing wire”*).

The venous ultrasound catheter is a simple ultrasound receiver catheter which acts as a target in the vein for aligning the needle of the arterial ultrasound catheter. The catheter tip features a 360° ultrasonic sensor which allows the catheter to detect the arterial ultrasound catheter at any circumference angle. The venous ultrasound catheter is a 5-Fr catheter with a usable length about 100-cm. The catheter is placed over a standard 0.014″ guide wire and is placed through a sheath in the femoral vein and advanced to the tibial vein up to and parallel to the arterial ultrasound catheter. The venous ultrasound Catheter is left in place and the arterial ultrasound catheter is rotated and moved longitudinally to obtain an optimal ultrasound signal indicating that the needle is aligned with and in the direction of the venous ultrasound catheter in the tibial vein.

Both arterial and venous catheters are intended to be used in a catheterization laboratory in consenting patients under fluoroscopy guidance. Both catheters are supplied sterile, removed at the conclusion of the procedure, and intended for single use only.

#### Valvulotome

The valvulotome is intended to make venous valves incompetent. The valvulotome is a device that is inserted over the crossing wire, passing the crossing section into the venous vessel. A push-pull deployment mechanism allows to deploy a nitinol cutting basket mounted at the distal tip. This cutting element self-centers in the venous vessel up to a maximum diameter of 4.5 mm. The actual cutting blades are arranged at a lower diameter as the maximum diameter of the cutting element, this prevents cutting the venous vessel but allows cutting of the vein vales ones the cutting element is pushed through the valves. The device (un-deployed cutting element) has a total outside diameter of 4 Fr. The valvulotome is supplied sterile and intended for single use only.

#### Stent grafts

To facilitate constant blood flow through the newly-created crossing from artery to vein, a stent graft needs to be inserted. The LimFlow stent grafts product line contains of different self-expanding stent graft sizes and shapes, in order to meet physiological variations in anatomy of patients, and one delivery system, which is compatible for each stent size. The blank laser-cut nickel-titanium alloy (nitinol) stent serves as basic for additional forming and electro spun PTFE encapsulation procedures to obtain final stent grafts.

The stent graft delivery system comprises of inner tubing, which serves as 0.018″ guide wire lumen and a flushing lumen, which is proximal accessible through a check valve. Design input specifications require 7-Fr sheath compatibility for the delivery device. In order to achieve excellent mechanical properties and functional push ability the outer shaft was designed with special braid pattern. Two radiopaque markers are attached to the distal end of the delivery device where the stent is crimped in between. Unintended stent movement during sheath retraction is restricted by the delivery device.

### Training and experienced needed

The procedure itself should be performed by vascular surgeons and/or interventionalists experienced in interventional techniques such as complex percutaneous transluminal angioplasty and stenting in the lower limb. Study investigators will be selected based on their experience in performing below-the-knee interventions and duly trained by LimFlow SA on the percutaneous deep vein arterialization specific medical procedure. All study investigators will be required to perform at least one (1) successful revascularization prior to participating to the study. In addition, a LimFlow SA representative will attend the first treatments performed in each site in order to assist the physician on technical issues as well as to ensure that treatment characteristics and potential complications are duly recorded.

### Medical procedures involved

The specific medical procedure involved in the use of the device is as follows:

1. Use sterile technique to carefully remove the catheters from the packaging. Inspect the catheters to verify that they are undamaged.
2. Obtain femoral artery access using standard Seldinger^1^ technique to place a 7-Fr introducer sheath.
3. Add tourniquet (pneumatic or Esmarch) above the knee to distend the veins and reduce the arterial flow.
4. Obtain tibial vein access using ultrasound-guided Seldinger^1^ technique with an echogenic 2.9/4.0-Fr micropuncture set and place the sheath at or above the level of the ankle. Dotter up to a 5-Fr × 45 cm sheath for the venous catheter.
5. Insert a 0.014″ guidewire through the arterial introducer sheath into the distal portion of the posterior or anterior tibial diseased artery. Advance the arterial catheter within a standard 7-Fr × 45/55 cm sheath in the artery.
6. Insert a 0.014″ guidewire through the venous introducer sheath and advance antegrade until it is above the corresponding tibial diseased artery.
7. Set-up the ultrasound system by connecting red and blue sterile electrical extension cables to (1) color-coded electrical connectors from arterial and venous catheters, respectively, and (2) ultrasound system.
8. Flush (female luer port) and introduce the venous catheter into the venous 5-Fr sheath over the wire. Advance the catheter into the venous system until it is parallel to the corresponding tibial diseased artery.
9. Remove the protection stylet wire at the tip out of the arterial catheter, flush the central lumen (female luer port at handle) using a syringe up to 5 ml. Preload the 0.014″ crossing wire in the arterial catheter. Advance the arterial catheter with the monorail 0.018″ guide wire into the femoral sheath to the distal arterial target parallel to the venous catheter. Align the arterial and venous catheters using the ultrasound system.
10. Once aligned, advance the arterial catheter crossing needle from tibial artery to tibial vein by deactivating the “twist lock” (turning the thumb piece clockwise and advancing in distal direction).
11. Insert the 0.014″ guidewire through the arterial catheter crossing needle and into the tibial vein going in a retrograde direction towards the foot.
12. Unplug the electrical connections from the ultrasound system.
13. Pull in the crossing needle by activating the “twist lock” (pulling thumb piece in proximal direction, thumb piece will flip back into lock position automatically). Ensure that the crossing needle is pulled back into the arterial catheter completely.
14. Remove the arterial catheter while leaving in place the guidewire going from the arterial target and into the tibial vein.
15. Advance a support catheter that accepts a 0.018″ guidewire through the arterial sheath in the tibial vein (pre-dilatation of the crossing area may be required) and advance wire and support catheter into and around the arch of the foot, anchoring the wire on the opposite tibial vein.
16. Exchange for a 0.018″ guidewire through the support catheter.
17. Insert the valvulotome in the 7-Fr arterial sheath and advance to the level of the foot.
18. Venous valves distal to the cross over point have to be made incompetent using the valvulotome to allow blood to flow to the distal part of the venous circulation of the foot.
19. Place the stent graft delivery system over the 0.018″ guidewire and advance through the arterio-venous crossing point.
20. Depending on patient anatomy, multiple stent grafts may be deployed. If not able to advance the first stent graft, remove the stent delivery system and advance a low profile over-the-wire PTA catheter to dilate the arterio-venous connection and place the stent delivery system again. The stented area should extend from the arterio-venous crossing area starting at the artery level and continuing into the tibial vein just above the ankle joint. Deploy stent grafts from distal to proximal, placing the crossing stent graph last. A minimum of 1-cm overlap is recommended between all placed stents grafts.
21. Once the stents are deployed, post-dilate extension and crossing stent grafts with a PTA catheter, choosing the diameter on the basis of the vessel size (3 to 6 mm).
22. Once the stent grafts are in place, the physician may decide to treat the inflow and/or outflow vessels.
23. Confirm placement of various catheters and stent grafts throughout the procedure under fluoroscopy using contrast injections.

Patients should receive adequate antiplatelet and anticoagulation therapy for a minimum of three (3) months post-procedure as per institution practice.

### Study outline

#### Screening and baseline

The subject screening and recruitment process will be performed by the site medical personnel and should follow the steps below:

1. Critical limb ischemia patients with no endovascular or surgical treatment options are initially identified by the site investigators
2. No-option CLI patients meeting the LimFlow System indications and contraindications (as provided in the instructions for use since the LimFlow System is commercially available) can be scheduled for a percutaneous deep vein arterialization
3. Patients scheduled for a LimFlow intervention are asked whether they would be willing to participate to the study (inclusion/exclusion criteria are aligned with the CE-marked indications/contraindications)
4. Patients candidate to the study are subsequently enrolled, treated, and followed as per standard of care (the study protocol does not require more visits than what is considered standard of care)

Once the patients have agreed to participate to the study (i.e., once the informed consent has been obtained), the following exams will be collected:

- Demographics, medical history, and peripheral assessment
- Infectious status, i.e., wound culture and/or blood analysis (WBC, CRP, ESR)
- Venous mapping of the foot, i.e., phlebography, duplex ultrasound, MRV, or CTV
- Arterial angiogram from the common femoral artery to the foot (two views)
- Serum creatinine level
- Rutherford classification
- WIfI (Wound – Ischemia – foot Infection) classification
- Wound pictures and assessment
- Pain assessment
- Perfusion assessment (TcPO_2_, ICG i.v. injection, white-light spectroscopy with laser Doppler, etc.—optional)
- Quality of Life (EQ-5D)
- Review of medications (antiplatelets/anticoagulants and antibiotics regimen)

**Fig. 1:**
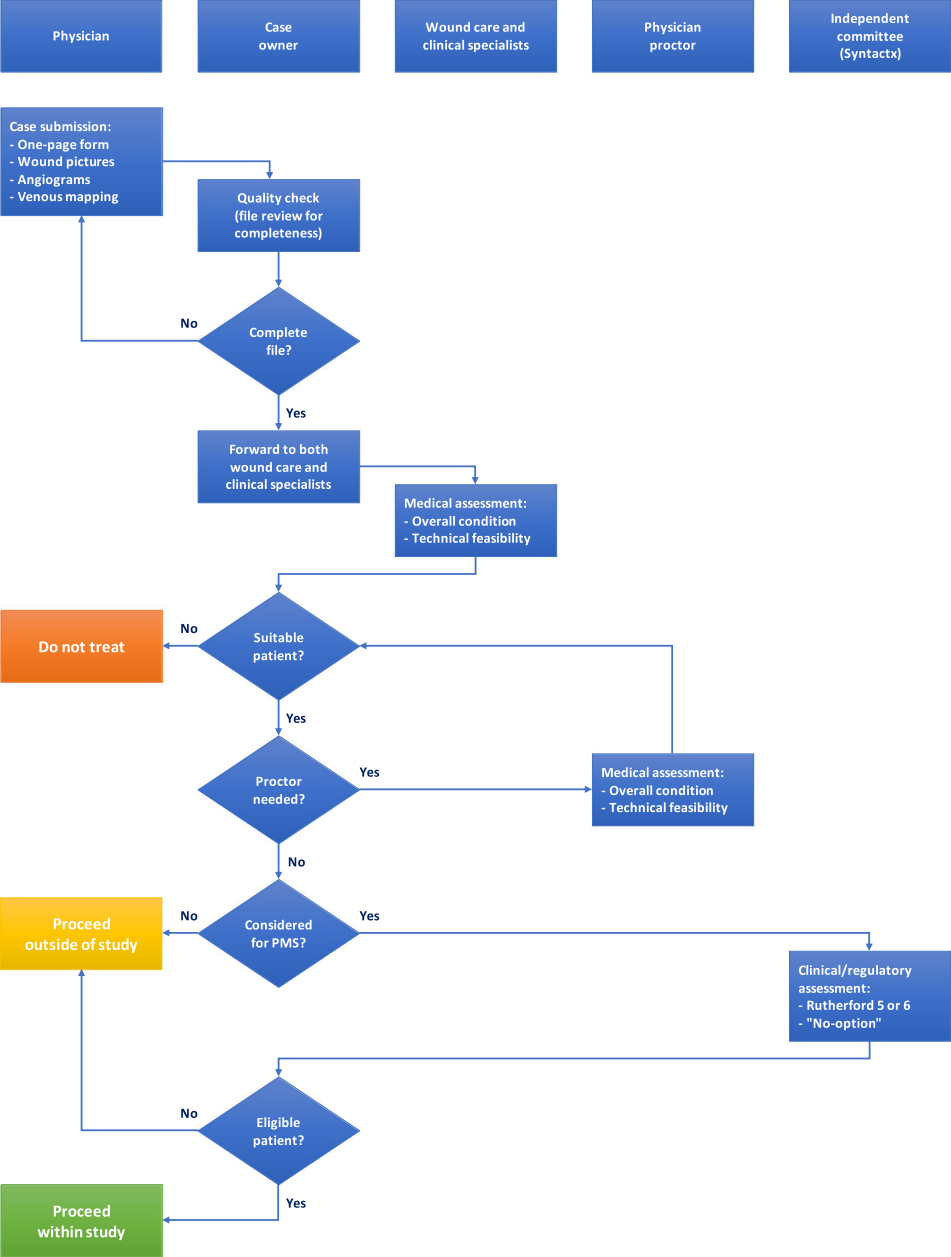
Eligibility screening process for LimFlow cases

A dedicated website (decidemedical.com platform provided by ClinFlows) will be used during the screening process to assess the eligibility of the candidates identified by the investigators. Baseline radiological images (e.g., arterial angiograms, phlebography, duplex ultrasound, MRV, or CTV) as well as wound pictures should be uploaded by the investigators in order for the patients’ eligibility to be confirmed. An independent committee will review the data and judge the eligibility for the patient to be included in the study. The eligibility screening process that will be followed for this post-market study is presented in detail on Fig. 1 below.

Timing for performing visits and assessing variables during the study follow-up period are presented in Tables 1 and 2 below. Additional post-treatment evaluations performed outside of the follow-up window will be considered “unscheduled”.

**Table 1:** Visits and exams Patients should be followed at regular intervals during one year after the initial percutaneous deep vein arterialization procedure in accordance with standard of care and local practice. Visits and exams scheduled for this post-market study are presented in 1 below (keys: ■ mandatory, □ optional)

Exams	Baseline	Treatment	Week 02	Month 01	Month 02	Month 03	Month 06	Month 09	Month 12
Eligibility	■								
Demographics	■								
Infection status^a^	■								
Venous mapping^b^	■								
Angiogram	■	■							
Ultrasound		Duplex		Duplex	Duplex	Duplex	Duplex	Duplex	Duplex
Medication	■	■	■	■	■	■	■	■	■
Creatinine	■			□			□		
Rutherford	■			■	■	■	■	■	■
WIfI	■			■	■	■	■	■	■
Wound pictures	■		■	■	■	■	■	■	■
Pain	■		■	■	■	■	■	■	■
Perfusion	□		□	□	□	□	□	□	□
Quality of Life	■					■	■	■	■
Adverse events		■	■	■	■	■	■	■	■

^a^Wound culture and/or blood analysis (WBC, CRP, ESR)

^b^Phlebography, duplex ultrasound, MRV or CTV, depending on what is available

**Table 2:** Time windows

Visit	Target	Interval	Minimum	Maximum
Two (2) weeks	15 days	± 5 days	10 days	20 days
One (1) month	30 days	± 7 days	23 days	37 days
Two (2) months	60 days	± 10 days	50 days	70 days
Three (3) months	90 days	± 14 days	76 days	104 days
Six (6) months	180 days	± 14 days	166 days	194 days
Nine (9) months	270 days	± 14 days	256 days	284 days
Twelve (12) months	360 days	± 28 days	332 days	388 days

All variables will be recorded on electronic Case Report Forms (eCRFs) specifically designed for the study and provided by LimFlow SA to the investigational centers. Collected variables will be analyzed using IBM SPSS statistics software, version 17 or later

Standard evaluation tools such as the Rutherford classification (Rutherford et al., 1997) or the SVS WIfI (Wound – Ischemia – foot Infection) classification system (Mills Sr. et al., 2014) will be used for the overall assessment of critical limb ischemia at baseline and follow-up visits. Pain will be evaluated using a numerical rating scale and the EuroQol (EQ-5D) questionnaire (EuroQol, 1990) will be used to assess the patients Quality of Life.

The eKare inSight (http://ekare.ai/) digital wound management platform (eKare, Inc., Fairfax, Virginia, United States of America) will be used for photographing, scanning, and assessing wounds at screening and follow-up visits. eKare inSight® is an FDA registered Class 1 medical device and is CE marked. Additionally, eKare is ISO 13485 certified and is fully compliant with FDA 21 CFR Part 820, Part 11.

### Endpoints

#### Primary endpoint

- Amputation-free survival

#### Secondary endpoints

- Complete wound healing
- Primary and secondary patency
- Limb salvage
- Renal function
- Technical and procedural success

#### Sample size calculation and date analysis

### Sample size

In the absence of formal statistical hypotheses for this single-arm, post-market study, the planned sample size could not be derived statistically. It was however estimated that a cohort of approximately fifty (50) subjects would provide sufficient data in order to meet the objectives defined in the study.

### Provision for an interim analysis

Interim analyses may be performed at any time if deemed necessary to fulfill the sponsor’s or manufacturer’s reporting requirements and/or update the evaluation of the side effects and of the acceptability of the benefit/risk ratio, as required in Council Directive 93/42/EEC of 14 June 1993 concerning medical devices.

### Specification of subgroups for analysis

If relevant, subgroup analyses may be performed based on baseline or treatment parameters—Student’s (or Wilcoxon’s according to the normality of the distribution) and Chi-2 (or Fisher’s) between-group tests will then be used for quantitative and qualitative parameters, respectively.

In particular, renal function characteristics at baseline (GFR value and/or dialysis status) may be used to define subgroups for the purpose of the analysis. Specifically, patients on dialysis are excluded from the early feasibility study (EFS), which will be taken into consideration when pooling datasets from both cohorts. Safety and effectiveness results from dialysis and non-dialysis patients will be compared.

### Risks and benefits

#### Anticipated clinical benefits

The patients designated for percutaneous deep vein arterialization have critical limb ischemia with no treatment option other than major amputation. These patients have had repeated percutaneous procedures to use angioplasty to open up the below-the-knee vessels, but the re-occlusion rate is high and once the foot is deserted (lack of blood circulation to the foot), ischemia and necrosis quickly set in. Necrotic tissue has to be cut away to allow healthy tissue a chance to heal. Infection is a major complication that can rapidly become systemic leading to mortality. Critical limb ischemia patients are desperate as all medical experts have told them that there are no more possible treatments. The lack of options highlights percutaneous deep vein arterialization is a last hope treatment for these patients, who have exhausted all other possibilities. For this reason, the benefits of percutaneous deep vein arterialization with the LimFlow System far outweigh the known risks associated with this device and readily available percutaneous angioplasty equipment.

### Anticipated adverse device effects

There is an independent medical monitor from Syntactx (NY) who will review all (severe) adverse events. The following adverse events are considered to be anticipated when performing any percutaneous catheterization:

- Allergic reaction, including anaphylactic shock and Quincke’s oedema
- Vascular complications at access site, including bleeding events and hematoma
- Arterial and venous thromboembolic events, including angina or myocardial infarction, stroke or transient ischemic event, pulmonary embolism, deep vein thrombosis, and limb ischemia
- Contrast-induced nephropathy and renal failure
- Local or systemic infection
- Pain

### Residual risks associated with the investigational device

The LimFlow System was reviewed in accordance with a risk management process that complies with the international standard on the application of risk management to medical devices EN ISO 14971:2012. The risk management process entailed an analysis of potential risks and an evaluation of their acceptability in the light of the intended therapeutic use of the system. The purpose of the risk analysis was to identify and characterize undesirable events that could result in harm. For each identified hazard the risks were estimated by factoring in the probability of occurrence and severity of the harm. The design verification report was reviewed to verify that all risk control measures identified as necessary to reduce risk to acceptable levels had been implemented and that no risks would arise from the implementation of control measures. It was concluded that all risks associated with the identified hazards had been reduced to acceptable levels and that the overall risk of the use of the LimFlow System was determined to be acceptable. The LimFlow System received CE-mark in October 2016.

### Ethics

This study is conducted in accordance with the principles of the Declaration of Helsinki and ‘good clinical practice’ guidelines. The Medical Ethical Committee of the NorthWest Clinics in Alkmaar, the Netherlands, has approved the protocol. The Ethical Committees of the participating centers is applied for local feasibility. Prior to randomization, written informed consent will be obtained from all patients.

## Discussion

Critical limb ischemia (CLI) is the end stage of peripheral artery disease (PAD) and is associated with high amputation and mortality rates. (Sprengers et al., 2010) The quality of life is poor and data of patients with no-option CLI is significantly worse than scores previously obtained in patients with cancer, chronic heart disease, and chronic kidney disease underlining the need for improved treatment of these patients. (Sprengers et al., 2010)

Bypass surgery and the last decade endovascular interventions with angioplasty and stenting have become the treatment of choice. The percutaneous revascularization represents a revolution in the treatment of CLI. Novel techniques have increased the number of limbs being salvaged. However, when there is an absence of distal target vessels or only small artery’s (the “desert foot”) it often leads to failure of all revascularization attempts and culminates in major amputation. These are the no-option CLI patients where a venous arterialization can be a viable alternative.

A meta-analysis of the venous arterialization in patients with no-option CLI, show a pooled limb salvage rate at 12 months of 75%. (Schreve et al., 2017) The last decade several studies have been published with promising results and the number of treated patients has considerably grown. Venous arterialization could be a valuable treatment option in patients with often no other options than amputation of the affected limb.

However, the “classic” venous arterialization has a downside with the surgical wounds on already critical foot. A major advantage of the pDVA is the minimal invasiveness with lower periprocedural risks and no creations of wounds in an already critically ischemic leg. The first results in men are promising although more research and long term follow up is needed to establish the efficacy of this new treatment modality. (Kum et al., 2017)

With this prospective study, we evaluate the clinical effectiveness and safety in patients with no-option CLI treated with the pDVA (LimFlow System).

## Data Availability

All data will be collected and analysed by the authors.

## Abbreviations

CLI: Critical Limb Ischemia
GFR: Glomerular Filtration Rate
PAD: Peripheral Artery Disease
pDVA: percutaneous Deep Vein Arterialization
WIFi: Wound – Ischemia – Foot Infection
